# Training and education in Digital Psychiatry: a perspective from Asia-Pacific Region

**DOI:** 10.1192/j.eurpsy.2022.2198

**Published:** 2022-09-01

**Authors:** L. Orsolini, U. Volpe

**Affiliations:** Unit of Clinical Psychiatric, Polytechnic University of Marche, Ancona, Italy, Department Of Neurosciences/dimsc, Ancona, Italy

**Keywords:** psychiatry training, training in digital psychiatry, digital psychiatry

## Abstract

**Introduction:**

Digital mental health interventions and digital psychiatry have been rapidly implemented over the past decade, particularly with the intent to offer a cost-effective solution in those circumstances in which the current mental health services and infrastructure are not able to properly accommodate the patients’ needs. However, mental health workforce is often poorly theoretical/practical trained in digital psychiatry and in delivering remote consultations safely and effectively, not being common to own curricula-specific training requirements in digital psychiatry and skills.

**Objectives:**

Our aim is evaluating the level of training, knowledge, experience and perception regarding the topic of digital psychiatry in a sample constituted by medical students, psychiatry trainees and early career psychiatrists from WHO South-East Asia and Western Pacific Regions (APAC).

**Methods:**

A web-based international cross-sectional survey was carried out to specifically investigating digita psychiatry in APAC regions.

**Results:**

An overall lack of theoretical and/or practical training on new digital tools and digital health interventions in psychiatry has been observed. The level of training influences knowledge background, which, in turn, influences young professionals’ perceptions and opinions regarding digital psychiatry and interventions in mental health.

**Conclusions:**

Implementing psychiatry training programs may significantly improve the level of knowledge and use of digital tools in mental healthcare. Moreover, mental health services and infrastructures should be properly adapted to the digital era, considering the overall weak and heterogeneous technical support and equipment, issues of internet connectivity and other administrative related challenges observed in APAC.

**Disclosure:**

No significant relationships.

